# Case Report: Massive intrapericardial hematoma following acupuncture therapy

**DOI:** 10.3389/fcvm.2024.1433945

**Published:** 2024-10-15

**Authors:** Weiguang Chen, Xiaoling Gao, Hong Li

**Affiliations:** ^1^ Department of Cardiology, 1st Hospital Affiliated of Hebei North University, Zhangjiakou, Hebei Province, China; ^2^ Department of Hyperbaric Oxygen Chamber, 1st Hospital Affiliated of Hebei North University, Zhangjiakou, Hebei Province, China; ^3^ Department of Cardiology, Beijing Chaoyang Hospital, Capital Medical University, Beijing, China

**Keywords:** acupuncture therapy, hematoma, cardiac tamponade, chest pain, syncope

## Abstract

Cardiac tamponade is a critical cardiovascular condition where timely diagnosis and treatment are crucial. The formation of an intrapericardial hematoma following acupuncture therapy is clinically rare. This paper reports a case of an elderly female patient who experienced severe chest pain and syncope during acupuncture therapy, subsequently diagnosed with traumatic hemopericardium and acute cardiac tamponade, complicated by cardiogenic shock. Under ultrasound guidance, pericardial puncture and drainage were successfully performed. The patient's symptoms were alleviated, her vital signs stabilized, and follow-up outcomes were favorable. This case provides valuable reference for understanding the pathogenesis, diagnosis, and treatment of pericardial hemorrhage following acupuncture therapy, integrating both clinical practice and literature review.

## Background

Pericardial disease and other conditions affecting the pericardium can lead to pericardial effusion. When the effusion accumulates rapidly or reaches a significant volume, it can substantially decrease venous return and cardiac output, potentially inducing cardiac tamponade. Hemorrhagic pericardial effusion, which can complicate into acute cardiac tamponade, may be caused by acute myocardial infarction, aortic dissection, trauma, cardiothoracic surgery, and interventional cardiac therapy. Acupuncture therapy, a key component of traditional Chinese medicine, involves inserting filiform needles into specific parts of the body at certain angles. These techniques, such as twisting and lifting, are used to stimulate targeted areas based on traditional Chinese medicine theory to treat various conditions. This paper presents a rare case of hemorrhagic pericardial effusion with intrapericardial hematoma following acupuncture therapy.

## Case presentation

**Figure 1:**
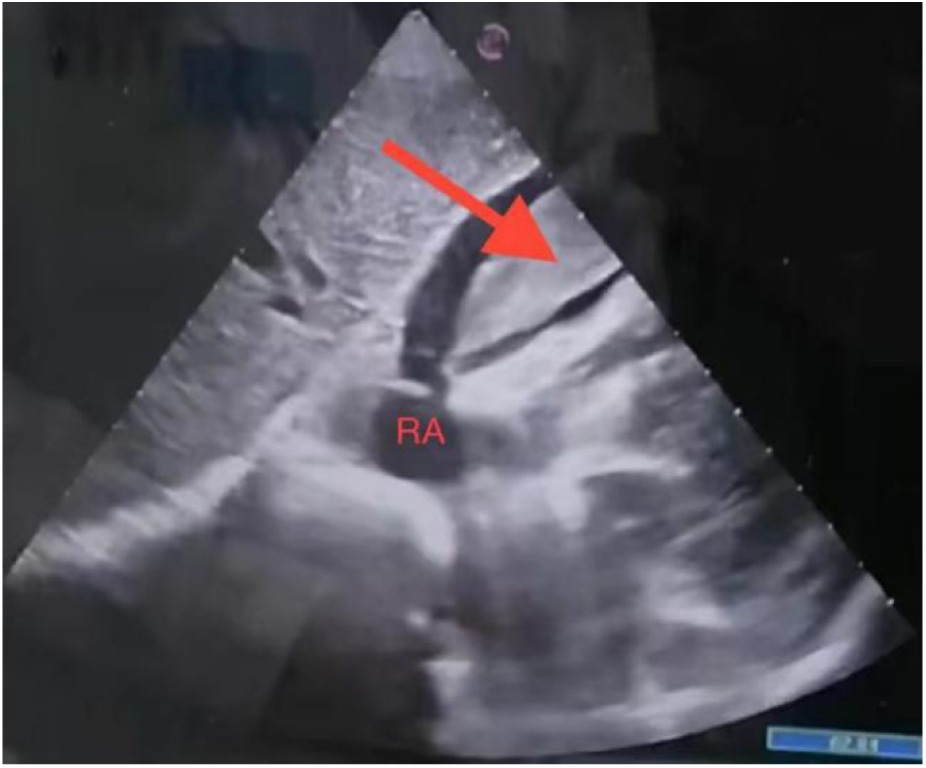
Subxiphoid four-chamber view: pericardial effusion and a mass were observed.

A 69-year-old woman was brought to the emergency department with complaints of chest pain and syncope. Eight hours prior, the patient had undergone acupuncture therapy at a local clinic to treat heart palpitations. The acupuncture was performed below the xiphoid process. Approximately ten minutes after the needles were inserted, the patient suddenly lost consciousness. Upon regaining consciousness, she reported severe chest pain, accompanied by dizziness, palpitations, sweating, nausea, and vomiting. She was then transferred to the nearest county hospital. Upon arrival at the county hospital, her vital signs were as follows: blood pressure 75/42 mmHg (1 mmHg = 0.133 kPa) and pulse rate 90 bpm. An echocardiogram, provided via video by her son, revealed a large mass within the pericardial cavity along with pericardial effusion (Figure 1). Despite intravenous dopamine infusion, the patient's symptoms of chest pain, dizziness, palpitations, sweating, and nausea persisted, and her blood pressure remained low at 85/66 mmHg. The patient denied any history of hypertension, diabetes, cardiovascular disease, and family history of Marfan syndrome. Additionally, there was no history of trauma or prior surgeries.

**Figure 2:**
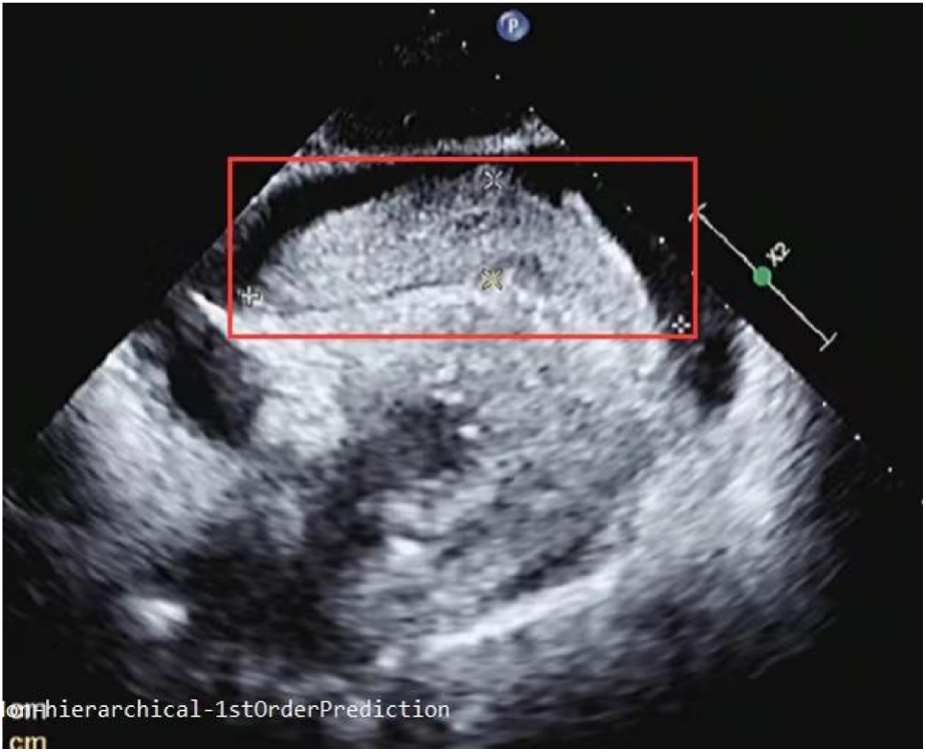
Subxiphoid four-chamber view: a giant cord-like soft tissue measuring 93 mm in length and 21 mm in width was observed in front of the right atrium and ventricle.

The patient presented with significant subxiphoid pain, dizziness, and nausea upon admission to the ward. Physical examination findings included: temperature of 36.6°C, respiration rate of 25 bpm, blood pressure of 95/65 mmHg, and a heart rate of 82 bpm. The patient was in an irritable state. Moist rales were detected at the bases of both lungs. Cardiac examination revealed weakened heart sounds at the apex, a slightly enlarged cardiac dullness boundary on both sides, a regular heart rhythm, and low heart sounds without murmurs or pericardial friction rub. The abdominal examination was unremarkable. Pulsus paradoxus was not observed. The patient's extremities were cold and clammy. An urgent ECG showed no abnormalities. Color Doppler ultrasound revealed a large mass of soft tissue floating in the pericardial cavity with a low to moderate effusion. There was no abnormal segmental wall motion, and left ventricular systolic function was normal (Figure 2).

**Figure 3:**
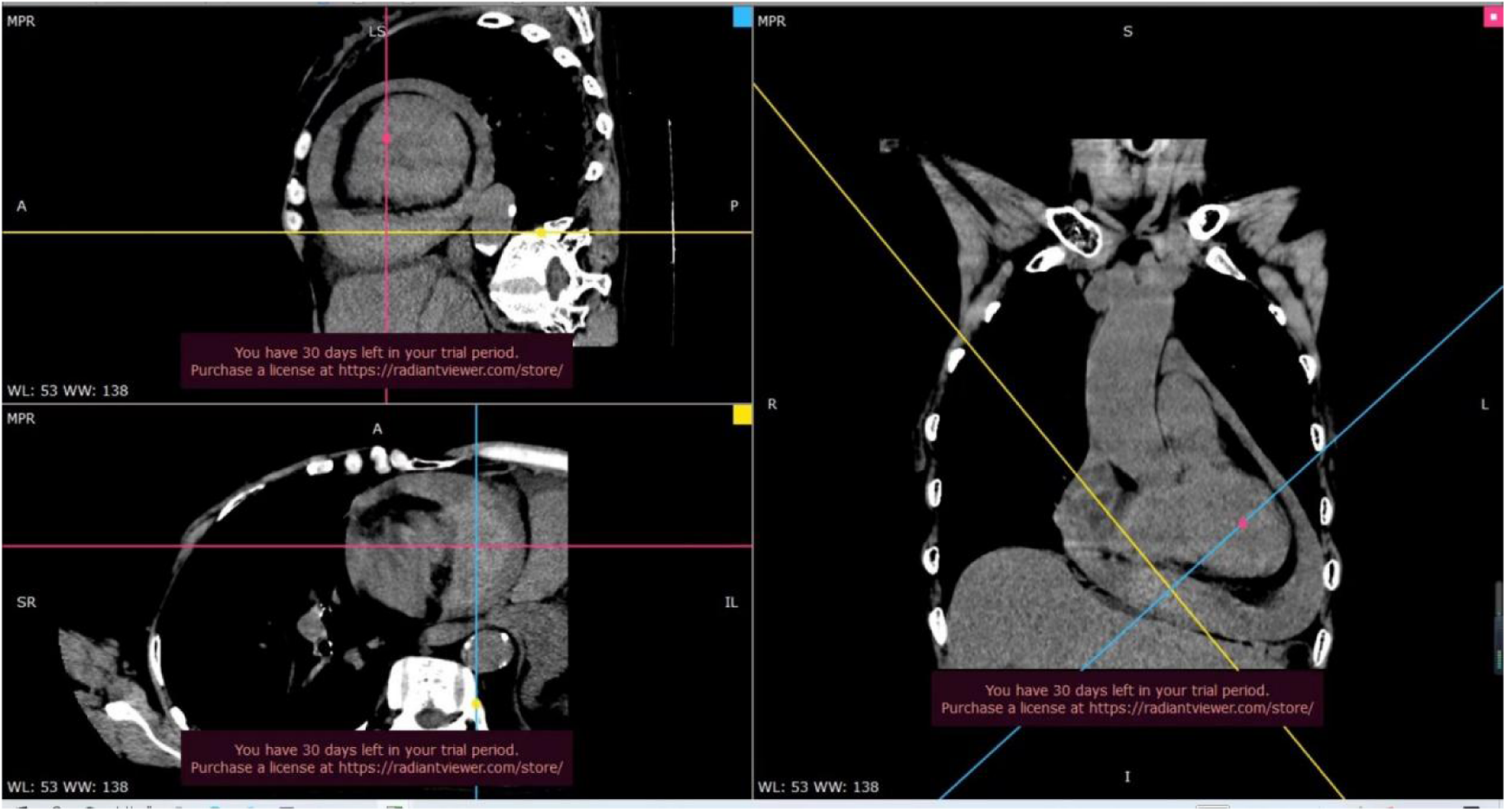
On thoracic aortic CT, a widespread slightly low-density shadow was observed in the pericardial cavity, with a CT value of approximately 30–36 hounsfield units (Hu), indicative of hemorrhagic pericardial effusion. Additionally, a mass tissue with slightly high-density shadow was noted in the pericardial cavity above the diaphragm, with a CT value of about 57 Hu. The boundary of this mass was unclear, and no significant enhancement was observed after contrast-enhanced scanning.

Thoracic aorta CT revealed hemorrhagic pericardial effusion, with no evidence of aortic dissection or cardiac rupture (Figure 3).

The patient's mental state remained very poor, with a blood pressure of 75/48 mmHg and a heart rate of 80 bpm. Arterial blood gas analysis indicated a pH of 7.25, PCO2 of 26 mmHg, PO2 of 127 mmHg (oxygen flow rate was 5 L/min), and a lactic acid level of 6.6 mmol/L. Liver function tests showed AST at 984 U/L (reference range: 0–35 U/L) and ALT at 411 U/L (0–35 U/L). The D-dimer was >10 mg/L (normal range: 0–0.5 mg/L), TnI was 0.21 ng/ml (0–0.3 ng/ml), and NT-proBNP was 2,018 pg/ml (normal range: 0–150 pg/ml). The admission diagnosis was acute cardiac tamponade with cardiogenic shock. Bedside echocardiography showed inferior vena cava dilatation, low to moderate pericardial effusion, and the pericardial apex was not suitable for puncture drainage. The pericardial effusion and soft tissue were primarily located anterior to the right ventricular wall. To identify the nature of the lesion, diagnostic pericardiocentesis was performed, yielding 4 ml of non-clotting blood, suggesting the mass could be an intracardiac hematoma. Subsequently, pericardiocentesis was performed via the subxiphoid approach, draining 450 ml of hemorrhagic effusion. The patient's blood pressure gradually increased to 145/83 mmHg, heart rate decreased to 61 bpm, and related symptoms significantly improved.

**Figure 4:**
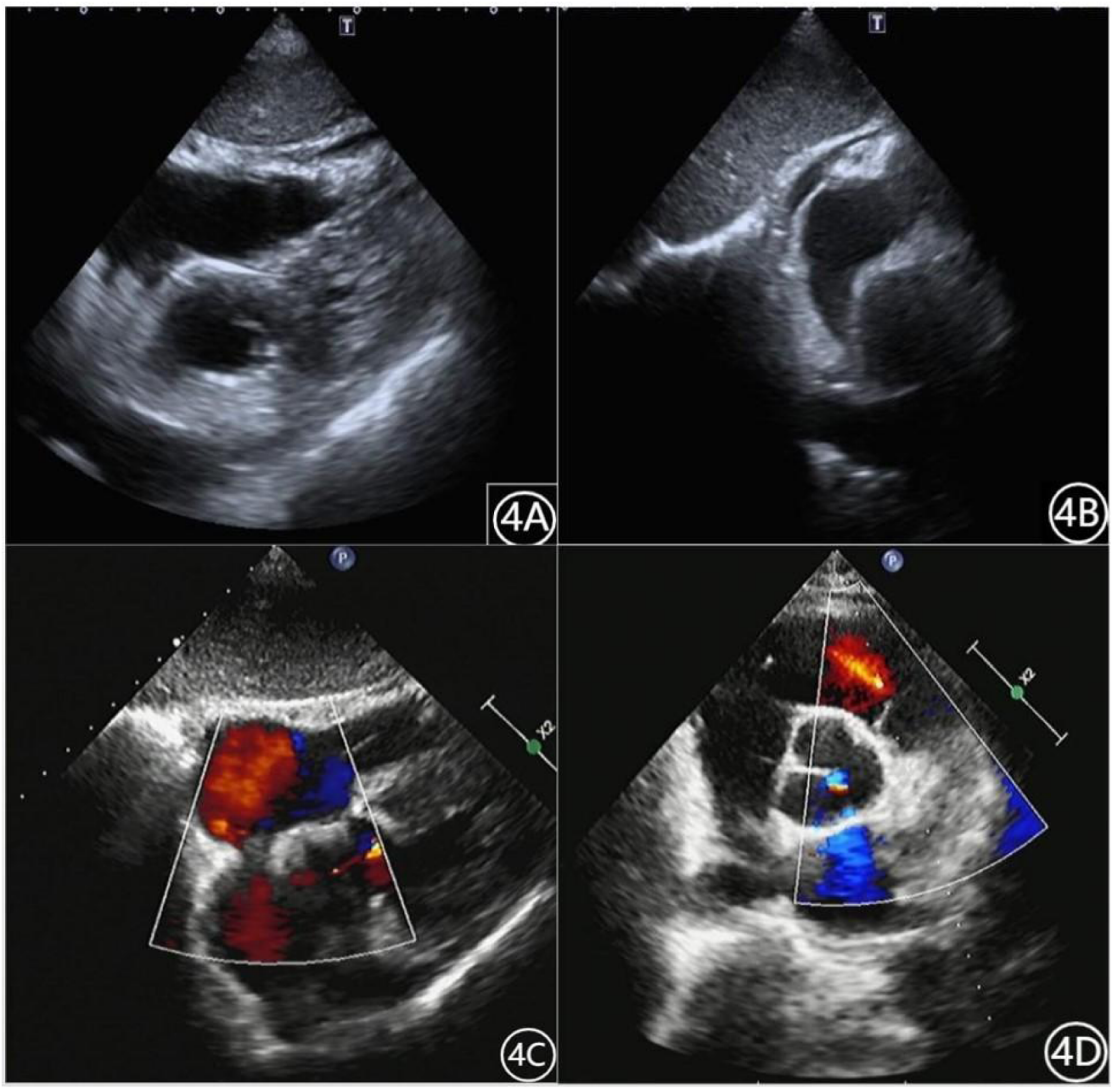
Patient's cardiac ultrasound data **(A)**: May 23, 2022. **(B)**: May 28, 2022. **(C)**: June 6, 2022. **(D)**: July 8, 2022.

On the third (Figure 4A) and eighth (Figure 4B) days of hospitalization, echocardiography showed minimal pericardial effusion and no pericardial masses. During the remainder of the hospital stay, the patient experienced no chest pain or palpitations and was discharged after removal of the drainage tube. Follow-up echocardiography on day 7 (Figure 4C), day 18 (June 18, outside hospital), and day 38 (Figure 4D) showed no significant pericardial effusion, and the patient reported no complaints during daily activities.

## Discussion

Hemorrhagic pericardial effusion can result from various causes such as puncture wounds, ventricular rupture, aortic dissection, cardiothoracic surgery, and coronary perforation during interventional procedures. Rapid or massive effusion can lead to pericardial tamponade, a critical condition that requires prompt intervention (1). This effusion is commonly associated with heart rupture, particularly following acute myocardial infarction, where most cases involve free ventricular wall rupture, and the mortality rate is exceedingly high. Studies indicate that approximately 90% of patients over the age of 60 with cardiac tamponade experience sudden death, often occurring within 1–5 days after myocardial infarction, with a significant number within the first 24 h (2). Aortic dissection results from the tearing of the intima and media layers of the aorta, leading to the entry of blood into the media through the tear. This process causes separation of the aortic wall into true and false lumens. Some patients with involvement of the ascending aorta may develop pericardial effusion, and severe cases may progress to pericardial tamponade. Studies have shown that acute circulatory failure due to cardiac tamponade is a primary cause of death in cases of acute aortic dissection rupture (3). Upon admission, the patient underwent emergency cardiac color ultrasound, which revealed no abnormal segmental wall motion, and the myocardial infarction test was negative. Additionally, thoracic aorta CT did not reveal any evidence of cardiac rupture or aortic dissection.

Acupuncture therapy is widely embraced not only for their efficacy but also for their relatively high safety profile and minimal complications. However, albeit rare, acupuncture-related pericardial tamponades can carry a mortality rate of up to 20% (4, 5). In acupuncture therapy, the Jiuwei point is situated in the upper abdomen along the front midline, approximately 1 inch below the xiphoid process. This point is primarily utilized to alleviate symptoms such as chest tightness, palpitations, heartburn, stomach pain, vomiting, and epilepsy. The procedure involves a direct stab of 0.3–0.6 in. or a downward oblique stab of 0.5–1 in. Generally, the depth of penetration for adults does not exceed 2.5 cm (6). It is recognized that improper use of acupuncture needles can lead to cardiac tamponade due to the proximity of the heart's anterior surface to the skin, typically ranging from approximately 13–19 mm. Even seasoned acupuncturists run the risk of inadvertently puncturing cardiac chambers or coronary arteries with acupuncture needles.

Several reports highlight the occurrence of acute pericardial tamponade as a complication of acupuncture therapy. In one case, subxiphoid acupuncture led to acute pericardial tamponade, necessitating emergency thoracotomy. The procedure revealed a hematoma on the anterior wall of the right ventricle with active bleeding (7). Another case involved acupuncture therapy performed at the left and right edges of the sternum at the 2nd and 3rd intercostal spaces. Subsequent heart color ultrasound revealed moderate pericardial effusion, with low to moderate degree strip echo observed outside the lateral wall of the left ventricle. Emergency thoracotomy unveiled a superficial myocardial tear of approximately 1 cm near the pulmonary valve ring in the right ventricular outflow tract, with a depth of less than 1 mm and slow blood exudation (8). Additionally, a puncture hole with active bleeding, measuring 2–3 mm in diameter, was discovered in the anterior wall of the right ventricle during thoracotomy exploration in another reported case (9). More recently, a case of acute cardiac tamponade occurred during the treatment of gastric ulcer at the Zhongting point. Following thoracotomy, a wound with a diameter of approximately 5 mm was observed at the bottom of the right atrium (10).

The cardiac ultrasound of this patient reveals the presence of soft tissue within the pericardium, which may float either to the left or right. Additionally, there is suspicion of a pedunculated structure between the right atrioventricular sulcus. It is essential to note that the right coronary artery runs along the coronary sulcus between the root of the pulmonary artery trunk and the right atrial appendage to the diaphragmatic surface of the heart. Therefore, needle puncture injury to the xiphoid process poses a risk of damaging the right coronary artery. In a reported case involving subxiphoid acupuncture, pericardial hematocele ensued. Thoracic exploration revealed a substantial right atrioventricular hematoma, accompanied by a broken branch vessel of the right coronary artery. Hemostasis was achieved by ligating the vessel during the procedure (11). Apart from thoracotomy for hemostasis, treatment strategies may also involve the utilization of covered stents for proximal lesions and spring coils for distal lesions. These interventions aim to effectively manage bleeding and prevent further complications (12).

Despite the stabilization of the patient's condition following pericardial puncture drainage and anti-shock treatment, and the decision to forego emergency chest exploration, this case presents several limitations. Firstly, while it is hypothesized that cardiac injury caused by acupuncture led to the formation of a hematoma and subsequent pericardial hemorrhage, the precise location of the puncture injury remains undetermined. Additionally, although cardiac ultrasound showed soft tissue in the pericardium, the nature of this tissue is unclear, making an exact diagnosis challenging. Further imaging or surgical exploration might have provided a more definitive assessment of the injury site. Moreover, ongoing close monitoring is essential to prevent recurrence.

## Conclusion

In conclusion, reports on cardiac tamponade caused by acupuncture therapy are not rare. But there are few reports on hematoma formation in the pericardial cavity. This patient underwent active non-surgical treatment, and the clinical outcome was good. Whereas there were some shortcomings in the diagnosis and treatment process. Firstly, there was a lack of understanding of the formation of pericardial hematoma following needle puncture injury. Based on the ultrasound video from the local hospital, the diagnosis was not made in time. Sequentially cardiac color ultrasound and aortic CT were performed for differential diagnosis, thus increasing the medical risk of the patient. Secondly, the understanding of acute cardiac tamponade is not impressive. Cardiac ultrasound has detected pericardial effusion, and combined with medical history and clinical manifestations of cardiac tamponade, pericardiocentesis was not performed as early as possible. Therefore, patients with a history of trauma should undergo urgent pericardial puncture and, if necessary, emergency chest exploration once they experience clinical manifestations of cardiac tamponade. Although this patient is relatively rare in clinical practice, it has a sudden onset, a dangerous condition, and a high mortality rate. Early diagnosis and treatment are crucial.

## Data Availability

The original contributions presented in the study are included in the article/Supplementary Material, further inquiries can be directed to the corresponding author.
